# Demography of some non-native isopods (Crustacea, Isopoda, Oniscidea) in a Mid-Atlantic forest, USA

**DOI:** 10.3897/zookeys.515.9403

**Published:** 2015-07-30

**Authors:** Elisabeth Hornung, Katalin Szlavecz, Miklós Dombos

**Affiliations:** 1 Department of Ecology, Institute for Biology, Faculty of Veterinary Science, Szent István University, H-1400 Budapest, P.O.Box 2, Hungary; 2 Department of Earth and Planetary Sciences, The Johns Hopkins University, 3400 N. Charles St., Baltimore, MD 21218-2681, USA; 3 Research Institute for Soil Science and Agricultural Chemistry of HAS, H-1525 Budapest, P.O. Box 102, Hungary

**Keywords:** Abiotic drivers, activity density, reproductive patterns, secondary sex ratio hypothesis, urban soil fauna

## Abstract

Introduced species dominate the terrestrial isopod fauna in most inland habitats of North America, including urban landscapes. These non-native species are often very abundant and thus potentially play a significant role in detritus processing. We monitored isopod assemblages in an urban forest for a year to examine the relationship between surface activity and abiotic environmental factors, and to analyze reproductive characteristics that might contribute to their successful establishment. Using pitfall trap samples we recorded five species, two of which, *Trachelipus rathkii* and *Cylisticus convexus*, were highly abundant. We determined size, sex and reproductive state of each individual. Surface activity of both species reflected variability in abiotic stress factors for isopods, such as soil moisture and soil temperature. Early spring the main trigger was soil temperature while later in the season increasing temperature and decreasing soil moisture jointly affected population dynamics. Activity significantly correlated with soil moisture. The temporal pattern of sex ratios supported the secondary sex ratio hypothesis. Males dominated the samples on the onset of the mating season in search of females. The pattern was reversed as females searched for suitable microsites for their offspring. Size independent fecundity decreased as conditions became more stressful late in the season.

## Introduction

In recent years there has been an increased interest in non-native, expansive soil invertebrates in North America. Studies almost exclusively focused on earthworm invasion (e.g. James and Hendrix 2004, Hale et al. 2005, Szlavecz et al. 2011a). Earthworms, as ecosystem engineers have multiple, profound and visible effects on soil physical and biogeochemical processes. However, the soil decomposer food web is complex, and other members of the fauna also contribute to these processes. Soil invertebrate community structure with special emphasis of non-native species other than earthworms, has received less attention, partially because their effects on ecosystem processes may be more subtle (Niemelä et al. 1997).

Terrestrial isopods are macro-decomposers that can significantly contribute to detritus processing (comminution, inoculation) and nutrient release. They occur also in habitats too extreme for earthworms, such as salt marshes, arid grasslands and deserts. Here and in other habitats they can reach extremely high local densities (e.g. Paris and Pitelka 1962, Steinberger 1976, Sorensen and Burkett 1977, Shachak et al. 1979, Dias et al. 2005, Messina et al. 2012) elevating them to the rank of the primary detritivore grazers and keystone group in regulating fungal communities (Crowther et al. 2013).

About one-third of the North-American Oniscidea is non-native. The endemic species mostly concentrate in coastal areas, caves, and the southern regions of the continent (Leistikow and Wägele 1999, Jass and Klausmeier 2000). Introduction of non-native woodlice, mostly from Europe, has been going on for centuries. Many of these species are synanthropic, and, probably due to lack of native fauna, successfully invaded wildland habitats, agricultural fields and cities. Isopods are among the most abundant arthropods in urban landscapes (Bolger et al. 2000, Smith et al. 2006, Vilisics et al. 2007a). Undoubtedly, life history characteristics of successful species at least partially explain their dominant status.

In this paper we report data on isopod demography in an urban forest in Baltimore, Maryland, USA. The study was part of a larger ongoing monitoring effort coordinated by the Baltimore Ecosystem Study (www.beslter.org, BES thereafter). BES is one of the two urban sites within the Long Term Ecological Research (LTER) network in the USA. One overarching question BES explores how heterogeneity in social, physical and biological factors interact to influence biodiversity (including soil biodiversity) at multiple scales (Swan et al. 2011, Szlavecz et al. 2011b). In the present study we examined the relationship between isopod population characteristics and abiotic environmental factors, and further analyzed reproductive characteristics that might explain high local abundance and thus invasion success of the dominant species.

## Methods

### Study site

We surveyed the isopod fauna in Leakin Park, a 492 ha contiguous parkland in Baltimore, Maryland, USA (39°15'N, 76°30'W). The park is about 8 km NW from the urban core, heavily forested, and surrounded with high density residential areas. The 90 year old forest belongs to the Tulip poplar Association (Brush et al. 1980) with common canopy species including Tulip poplar (*Liriodendron tulipifera*), several oaks (*Quercus alba*, *Quercus coccinea*, *Quercus velutina*), and American beech (*Fagus grandifolia*). Oaks and Tulip poplar make up 77% of the total annual litter mass, which is 4122 kg ha^-1^ (Groffman et al. 2006). The soil belongs to the Legore series (fine-loamy, mixed, mesic, Ultic Hapludalf). Duff layer is thin (0–2 cm), bulk density and pH of A horizon are 1.12g cm^-3^, and 5.1, respectively. More detailed description of the vegetation and soils is given in Groffman et al. (2006).

The climate can be characterized by hot humid summers and cold winters with average annual air temperatures ranging from 14.5 °C in the inner urban areas to 12.8 °C in the surrounding rural areas. Precipitation is distributed evenly throughout the year in the region and ranges from an annual average of 106.8 cm in Baltimore to 103.1 cm in the surrounding metropolitan area (NOAA, www.nws.noaa.gov).

### Sampling and laboratory measurements

Terrestrial isopods were sampled using pitfall traps (250 ml plastic cups) filled with propylene glycol. Ten traps were placed randomly around a 40 m × 40 m permanent forest plot established by the Baltimore Ecosystem Study LTER (Groffman et al. 2006). Traps operated between October 1999 and November 2000 and were emptied monthly except in late fall-winter when surface activity is generally low. The material was stored in 70% ethanol. All individuals were identified to species level using the nomenclature by Schmalfuss (2003).

Population and reproductive characteristics were determined only for the two abundant species. Because pitfall trap samples indicate a combination of surface activity and abundance of epigeic invertebrates, obtaining even relative density information creates a challenge. Recently, the term ’activity-density’ has been used (Melbourne 1999, Westerman et al. 2008) to express abundance, and here we follow this practice. Activity density is expressed as number of individuals caught per trap per day.

We estimated body size by measuring the widest point of the head capsule (cephalon) at the level of the eyes (Sutton 1968). Measurements were taken to 0.01 mm accuracy under a dissecting stereo microscope. Adult females were divided into three reproductive categories: non-reproducing, gravid (either with eggs, embryos or mancas), and post-reproductive (with empty marsupium). Reproductive period was defined as the time span between the appearance of the first gravid females and that of the last one with brood pouch (marsupium) either with or without progeny (Sutton et al. 1984).

### Abiotic factors

We obtained soil temperature and moisture data from the Baltimore Ecosystem Study database. Soil temperature was measured continuously using HOBO H8 Pro Series Temp/External Temp data loggers at 10 cm depth. For soil moisture measurements six time domain reflectometry (TDR) waveguide probes (Soil Moisture Equipment Corporation) were installed vertically into the soil at random locations throughout the plot. The waveguide probes are 20 cm long, so those vertically installed span a depth of 0 to 20 cm below ground. Soil moisture was measured once every four to six weeks.

### Statistical analysis

Mean daily values were used to explore correlation between soil temperature and activity density. Distance-weighted least squares fitting was used for smoothing. Due to lack of continuous soil moisture data, relationships between activity density and soil moisture was explored by using Spearman Rank Order Correlation Coefficient. We used multiple linear regression to explore relationships among fecundity (number of eggs produced by females), body size (cephalon width) and sampling date for each species. For computing these analyses we used STATISTICA 12 software (StatSoft Inc. 1984–2013).

To compare relative importance of independent variables i.e. size and time, we used standardized beta partial regression coefficients. Beta coefficients are obtained by setting all the variables to a mean of 0 and standard deviation to 1.

Ratio of males is expressed as the total number of males (M) over total number of adults (N) caught during a given trapping period (Dangerfield and Telford 1994). We conducted two different statistical tests. First, we wanted to estimate the unknown sex ratio and its uncertainty. Second, we wanted to assess the probability of the null hypothesis that the observed data is consistent with a M:N ratio of 0.5. A simple chi-square test informs only of the latter, moreover, chi-square test assumes Gaussian distribution i.e. symmetric error bars. This approximation is only valid when N is large. Since several of our samples were small, we chose a different approach (Gotelli and Ellison 2004). At a given sex ratio the distribution of observed M males out of a sample of N individuals (M<N) follows a binomial distribution. We first calculated the cumulative probability (CDF) that the number of males in the sample is equal or less than the measured count M. If M is more than half the sample size we estimate the probability that the number of males is equal or more than the count. Using this cumulative binomial distribution, we first computed the 95% confidence intervals in both directions, which often are not symmetric (Table 1). Finally we determined the probability the observed number of males is consistent with a sex ratio of 0.5, by simply reading off the midpoint value of the CDF.

**Table 1.** Abundance of *Trachelipus rathkii* and *Cylisticus convexus* between October 1999 and November 2000 in Leakin Park urban forest, Baltimore, USA. Numbers from all pitfall traps are pooled for each sampling period. Male ratio was calculated as proportion of males in total sample. 95% confidence intervals for these estimates are given the parentheses. Bold letters indicate significant differences from the expected 0.5 value.

Species	Month	Males	Females	Ratio of males	*p*
*Trachelipus rathkii*	October	110	118	0.48 (0.43; 0.54)	0.321
	November	38	61	**0.38** (0.31; 0.47)	**0.013**
	January	2	2	0.50 (0.25; 0.91)	0.312
	March	9	25	**0.26** (0.17; 0.42)	**0.004**
	April	76	58	**0.57** (0.50; 0.64)	**0.050**
	May	462	445	0.51 (0.49; 0.54)	0.275
	June	244	306	**0.44** (0.41; 0.48)	**0.005**
	July	401	461	**0.47** (0.44; 0.50)	**0.022**
	August	141	230	**0.38** (0.35; 0.43)	**< 0.001**
	September	90	128	**0.41** (0.37; 0.48)	**0.006**
	October	16	21	0.43 (0.32; 0.58)	0.256
	November	2	4	0.33 (0.15; 0.73)	0.343
*Cylisticus convexus*	October	185	252	**0.42** (0.39; 0.47)	**0.001**
	November	38	55	**0.41** (0.34; 0.50)	**0.048**
	January	0	0	NA	NA
	March	4	8	0.33 (0.18; 0.62)	0.194
	April	11	20	0.35 (0.24; 0.52)	0.075
	May	178	315	**0.36** (0.33; 0.40)	**< 0.001**
	June	263	353	0.51 (0.48; 0.55)	0.298
	July	352	568	**0.38** (0.36; 0.41)	**< 0.001**
	August	353	163	**0.61** (0.57; 0.65)	**< 0.001**
	September	162	227	**0.42** (0.38; 0.46)	**0.001**
	October	48	38	0.56 (0.47; 0.65)	0.117
	November	6	16	**0.27** (0.16; 0.47)	**0.026**

## Results

During the course of sixteen months a total of 2480 isopods were caught. The following five species were recorded: *Haplophthalmus danicus* Budde-Lund, 1880, *Hyloniscus riparius* (C. Koch, 1838), *Philoscia muscorum* (Scopoli, 1763), *Trachelipus rathkii* (Brandt, 1833) and *Cylisticus convexus* (De Geer, 1778). The pitfall material was dominated by the latter two species, with 1270 *Trachelipus rathkii* and 1073 *Cylisticus convexus* (53 and 45 %) individuals, respectively. Detailed analysis of the population characteristics is given only for these two species.

### Surface activity, demographic changes

**Figure 1.**
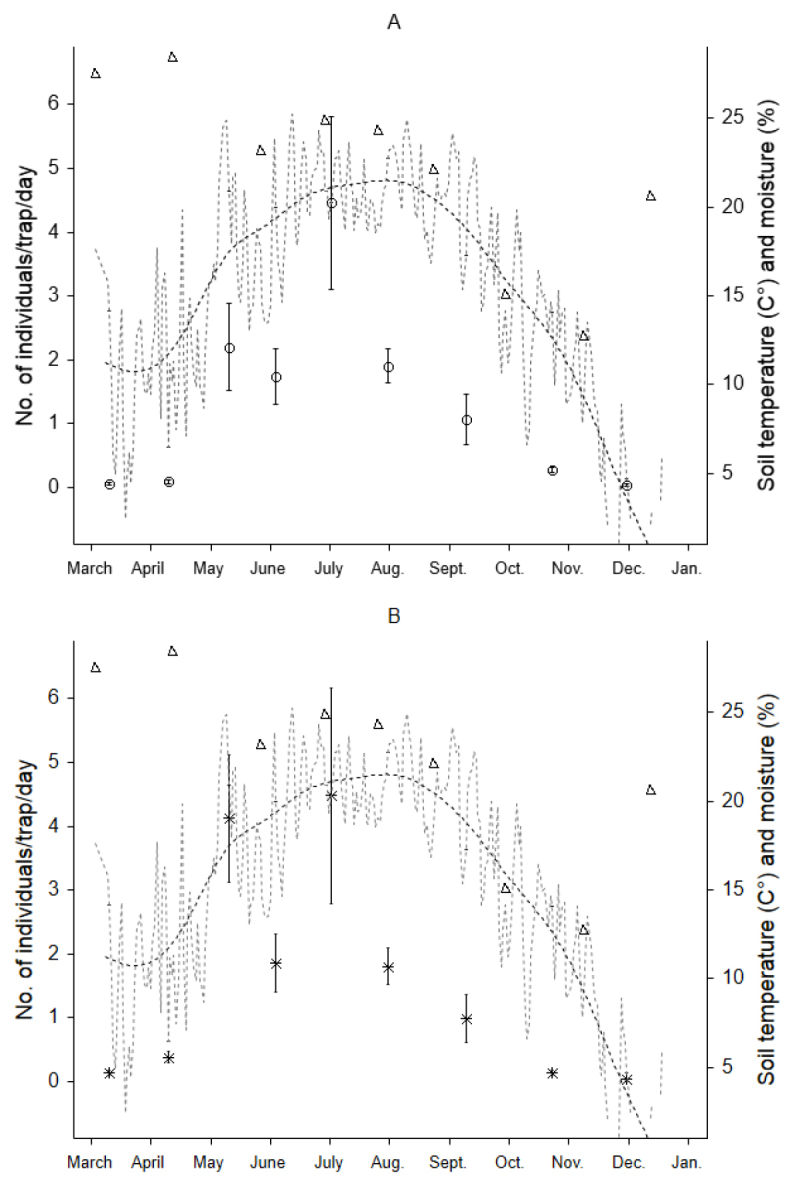
Temporal changes in activity-density of *Cylisticus convexus* (**A**) and *Trachelipus rathkii* (**B**) and soil physical characteristics in an urban forest in Baltimore. Mean numbers of individuals captured daily per trap (*Cylisticus convexus*: open circles, *Trachelipus rathkii*: asterix) ± SE are shown. Dotted line: mean daily soil temperature at 10 cm; dashed line: smoothed soil temperature; open triangles: volumetric soil moisture content.

Isopod numbers began to increase late April, peaked in July and declined in September and ceased by November (Fig. 1A and B). Activity density of both species positively correlated with soil temperature (r = 0.75, and r = 0.87, for *Trachelipus rathkii*, and for *Cylisticus convexus*, respectively). Soil moisture decreased during the growing season (Fig. 1A, B). Excluding winter months, when no isopods were caught, isopod activity positively correlated with soil moisture during the growing season (Spearman R = 0.32, *p* < 0.05 for *Trachelipus rathkii*, and R = 0.35, *p* < 0.05 for *Cylisticus convexus*).

### Reproductive characters

**Male ratio**

Male ratio varied over time, with the highest and lowest male : total ratio being 0.57 and 0.26 for *Trachelipus rathkii*, 0.61 and 0.27 for *Cylisticus convexus*, respectively (Table 1). For *Trachelipus rathkii*, we detected significant deviations from the expected the 0.5 ratio in seven sampling dates. In all but two months (April, May) activity density of females exceeded that of males. For *Cylisticus convexus* we detected significant differences also in seven cases. Again, only two months did males exceed females, but this happened later in the season (August, October).

**Reproductive period, phenology and fecundity**

*Trachelipus rathkii* started reproducing late April – early May (Table 2) with 20% of the females being gravid, and all in the egg carrying stage. Proportion of gravid females peaked at 57% in June–mid-July and slightly declined (47%) by mid-August. Gravid *Cylisticus convexus* females appeared in the traps later in the season (June–July), but at this period females both with eggs and empty marsupium were present. In September only a single gravid *Trachelipus rathkii* was caught, while 16% of the 146 female *Cylisticus convexus* in the sample were still gravid, all in post-reproductive stage.

**Table 2.** Percentage of reproductive *Trachelipus rathkii* and *Cylisticus convexus* in Leakin Park, Baltimore. Two stages are distinguished. Gravid: with eggs, embryos or mancas in the marsupium; postreproductive: empty marsupium.

			May	June	July	Aug
*Trachelipus rathkii*	All reproductive*		20.6	57.1	47.1	0.6
		Gravid**	100	41.7	17.5	0
		Postreproductive**	0	58.3	82.5	100
*Cylisticus convexus*	All reproductive*		0	61.0	40.0	16.0
		Gravid**	0	29.4	57.1	0
		Postreproductive**	0	70.6	42.9	100

* Calculated as percentage of all females in the sample

** Calculated as percentage of all reproductive females in the sample

**Figure 2.**
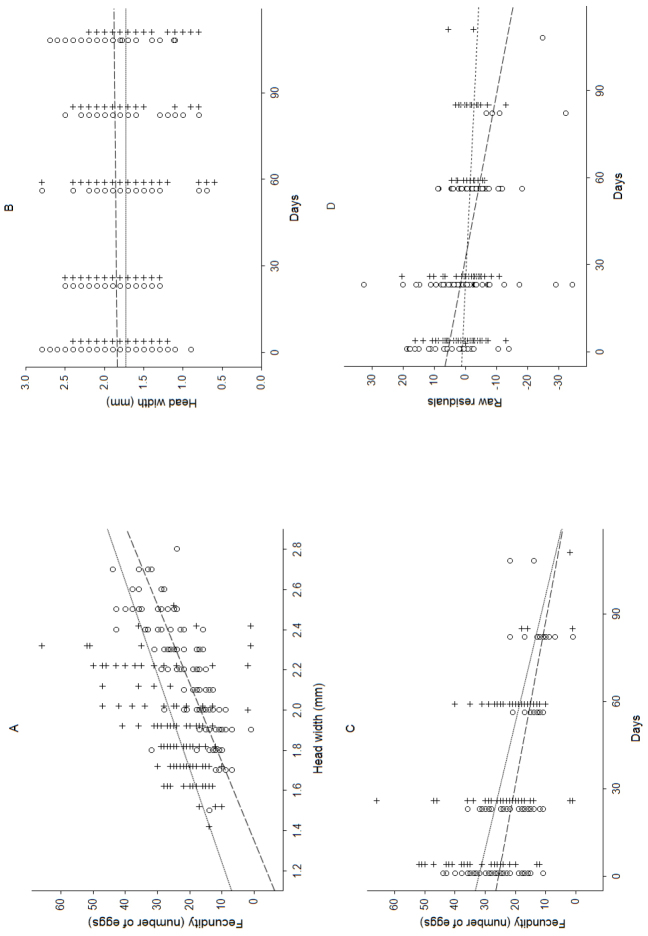
Fecundity of the dominant isopod species in Leakin Park, Baltimore. **A–B**: Relationship between fecundity and size (**A**) and its stability over time (**B**) **C–D**: Change of fecundity over time (**C**) and size independent fecundity over time, based on the residuals of egg numbers (**D**). *Cylisticus convexus*: open circles and dashed lines; *Trachelipus rathkii*: crosses and dotted lines.

Fecundity of females in *Cylisticus convexus* and *Trachelipus rathkii* were compared over time. We analyzed the relationship between fecundity and body size using multiple linear regression models: clutch size (number of eggs), as dependent variable, and body size (head width) and time (days from the start of the investigation), as independent variables. Regression summary for *Cylisticus convexus*: adjusted R^2 ^= 0.65, F(2,119) = 112.10, *p* < 0.001; beta(day) = -0.44; beta(head width) = 0.37. Regression summary for *Trachelipus rathkii*: adjusted R^2 ^ = 0.38, F(2,123) = 38.836, *p* < 0.001; beta(day) = -0.22; beta(head width) = 0.65. Number of eggs increased with body size (Figure 2A), and these relationships were stable throughout the season (Figure 2B). Size independent fecundity decreased with time for both species (Figure 2C): residuals of egg numbers showed negative trends, however, their slopes were different (Figure 2D). Standardized beta partial regression coefficients were -0.44 (p < 0.001) and -0.22 for *Cylisticus convexus* and *Trachelipus rathkii*, respectively, indicating that the decrease of female fecundity during the season was not linked to body size.

## Discussion

### Species composition

In the Greater Baltimore Metropolitan Area we have recorded a total of eleven terrestrial isopod species (Hornung and Szlavecz 2003). All species are non-native, and most have been known from North America for a hundred years (Hatch 1947, van Name 1936, 1940, 1942, Eberly 1953, Lindroth 1957, Jass and Klausmeyer 2000). Urban fauna is often characterized by synanthropic, generalist species that may lead to higher community similarity among cities (McKinney 2006). All five species present in Leakin Park fit this category, and the two dominant species *Trachelipus rathkii* and *Cylisticus convexus*, are among the ten most abundant exotics in North America (Jass and Klausmeier 2000). Both species are characterized as expansive in Europe and have been introduced to other continents, as well (Gruner 1966). In Europe both species occur in a variety of habitats, including cities (Korsós et al. 2002; Hornung et al. 2007, 2008, Vilisics and Hornung 2009, Vilisics et al. 2007a,b). The difference between their occurences in the two continents is that *Cylisticus convexus* tends to be more synanthropic in Central and Western Europe, its abundance is moderate to low, and is scarce or missing in the North (Gruner 1966, Berg et al. 2008, Vilisics et al. 2007a). In North America both species are widely distributed, and abundant populations of *Cylisticus convexus* and *Trachelipus rathkii* were reported e.g. from Michigan (Hatchett 1947) and Wisconsin (Jass and Klausmeier 1996), too.

### Temporal patterns

Temperature and relative humidity are known to be the main drivers of terrestrial isopod activity (Warburg 1987, 1993). Our data support this statement, but also show a more complex relationship in the field. Soil temperature is the main factor triggering surface activity early spring; however, later in the season increasing temperature and decreasing soil moisture jointly affect dynamics of the populations. In early fall even though temperature remains high, activity decreases (Fig. 1). At this time soil moisture is low due to high evapotranspiration rates by trees and lack of precipitation (Groffman et al. 2006). We acknowledge that other factors, such as size, mobility, and behavior may bias pitfall trap samples. However, the huge number of individuals in the samples gives us confidence that the dynamics we detected for these two abundant species, is real.

### Reproductive characteristics

**Male ratio**

With the exception of parthenogenetic species, where males occur in extremely low numbers, the sex ratio of most isopod species can be described by bimodality (e.g. Paris and Pitelka 1962, Sorensen and Burkett 1977, Hornung 1989, 1991). There are some examples for species with very different and constant adult sex ratio, e.g. *Porcellio ficulneus* Budde-Lund, 1885 male: female = 1:9 (Warburg 2007), and *Schizidium tiberianum* Verhoeff, 1923, 1:6 (Warburg and Cohen 1991). Sex ratio for terrestrial woodlice is routinely reported in papers focusing on population characteristics. In most cases these values are only snapshots obtained from samples of a short time period and do not reflect temporal changes of the sex ratio. Sex ratio might differ seasonally depending on different mortality and/or activity of sexes. We are aware of only a few long term studies where populations were frequently sampled to obtain changes of sex ratio over time (e.g Paris and Pitelka 1962, Sorensen and Burkett 1977). It is therefore important to excercise caution when comparing data for different populations even for the same species, as variations both in space and time may occur. Montesanto et al. (2008) reported a special case for *Platyarthrus aiasensis* Legrand, 1954 comparing 19 populations in and around Sicily. The populations differed in male ratio from 0 (parthenogenetic) to 0.37 of males.

Deviation from the expected 0.5:0.5 ratio may be due to behavioral differences between the sexes especially during reproductive period as proposed by the secondary sex ratio hypothesis (Dangerfield and Hassall 1994). According to this hypothesis at the onset of the mating season males are more active looking for receptive females. Later, gravid females exceed males in the sample, as they are looking for favorable microhabitats that maintain optimal conditions for their progeny. Both *Trachelipus rathkii* and *Cylisticus convexus* follow this pattern in our study site; the difference between them is the peak of timing of male searching behavior. High male dominance (0.98) was found also for *Protracheoniscus politus* (C. Koch, 1841) in Hungary (Oberfrank et al. 2011) and for *Armadillidium vulgare* (Latreille, 1804) in Texas (Sorensen and Burkett 1977) at the beginning of the activity season, before the onset of reproduction.

Gruner (1966) compiled and qualitatively reported population data for the two species we studied here. The percentage of *Cylisticus convexus* males was found to be lower than 50% in Denmark, France and Italy. For *Trachelipus rathkii* a female predominance was also reported.

**Reproductive period and phenology**

In Europe the onset of the reproductive period varied with latitude for both *Cylisticus convexus* and *Trachelipus rathkii* (Gruner 1966). For instance, *Cylisticus convexus* started reproducing in April in France; in Denmark the onset shifted to June. The number of offspring ranged between 14–50 per female, with extremely high numbers (73 embryos per female) in Italy (Gruner 1966). *Trachelipus rathkii* was reported to have two broods between May and September.

Jass and Klausmeier (2004) found a latitudinal difference in reproductive peaks in North America for several species including the ones studied here. In Wisconsin gravid females of *Cylisticus convexus* were present during June-July in the populations while reproductive period of *Trachelipus rathkii* was longer (June-August). In the present study the reproductive period started earlier, in April both for *Trachelipus rathkii*, and for *Cylisticus convexus*, and ended in August and September, respectively.

**Reproductive output over time**

Reproductive output is an important component of life history strategies and has a cost of decreased parental survival (Stearns 1976). The strong correlation between female size and egg number in terrestrial isopods is well known (e.g. Dangerfield and Telford 1990, Ma et al. 1991, Nair 1978, Warburg 2011). However, females may not allocate the same amount of energy into reproduction during the entire season. We found that the mean number of eggs/embryos decreased independently of female size as the reproductive period progressed. This tendency is the consequence of increasing environmental stress (unfavorable humidity and temperature changes, drought) and contributes to the costs of survival (Cody 1966, Charnov 2002, Hornung and Warburg 1993, 1994, 1998). Isopods can either invest less or regain part of the energy invested in reproduction earlier by reabsorbing ovarian oocytes or marsupial eggs under stressful conditions (Hornung and Warburg 1993, 1994).

**Successful establishment, expansion, invasion**

There is still some confusion regarding terminology in invasion ecology. The term invasive species is used for species “exhibiting rapid spread, irrespective to impact” (Davis 2009), as well as for species showing “demonstrable ecological or economic impact” (Lockwood et al. 2007). The two isopod species we reported here undoubtedly fit the first description, as both are well established and common throughout North America (Jass and Klausmeier 2000). The main characteristics successful invaders share include high dispersal rate, high genetic variability, short generation time, large number of offspring, broad diet and ecological tolerance. Some of these traits overlap with what Sutton et al. (1984) describes for “eurodynamic”, essentially *r* strategist species. Detritivores are by definition resource generalists (even though they have food preferences) allowing them to find food in a variety of habitats. Both *Cylisticus convexus* and *Trachelipus rathkii* have high fecundity, and can reproduce several times per season. Moreover, female isopods have been shown to be able to store sperm and utilize their stock in repeated reproduction events (Suzuki and Ziegler 2005). This can be especially significant when only one or a few females are transported from one habitat to another. Isopods are relatively easy to transport with soil, mulch, ornamental plants, timber and other means. Once introduced, they can quickly establish and, being mobile epigeic species, further disperse on their own. Garthwaite et al. (1995) estimated that *Armadillidium vulgare*, another common species in North America spread across the continent and reached the West Coast in about 150 years. We can only speculate that the lack of native relatives or ecologically equivalent soil fauna further facilitated the spread of terrestrial isopods including the species studied here.

The highly altered urban habitats can serve both as points of introduction via trade or transportation, and refuges for non-indigenous soil fauna. Residential areas provide food (e.g. compost, mulch) and shelter (building foundation, landscaping objects), while green corridors or even underground pipe systems can be conduits for dispersal. High epigeic isopod abundance has been repeatedly shown in urban habitat fragments and suburbs (Bolger et al. 2000, Smith et al. 2006, Vilisics et al. 2007a). Often, the same species dominate urban isopod fauna. *Trachelipus rathkii* and *Cylisticus convexus* belong to this successful cosmopolitan group, contributing to the global phenomenon of biotic homogenization in cities (Perrings et al. 2010).

## Conclusions

We examined population dynamics and reproductive characteristics of terrestrial isopods in an urban forest in Baltimore, Maryland, USA. Temporal patterns of male ratio supported the secondary sex ratio hypothesis for both dominate species. As expected, fecundity was correlated with female size. However, size independent reproductive output declined during the active season indicating a response to increasing stress. High fecundity, good dispersal ability and broad habitat and resource tolerance all may contribute to the invasion success of the investigated species in North America. Additionally, lack of native competitors and locally favorable conditions can further facilitate their spread and persistence in many ecosystems.
